# Feedback activation of AMPK-mediated autophagy acceleration is a key resistance mechanism against SCD1 inhibitor-induced cell growth inhibition

**DOI:** 10.1371/journal.pone.0181243

**Published:** 2017-07-13

**Authors:** Akito Ono, Osamu Sano, Ken-ichi Kazetani, Takamichi Muraki, Keisuke Imamura, Hiroyuki Sumi, Junji Matsui, Hidehisa Iwata

**Affiliations:** 1 Biomolecular Research Laboratories, Pharmaceutical Research Division, Takeda Pharmaceutical Company Ltd., Fujisawa, Kanagawa, Japan; 2 Oncology Drug Discovery Unit, Pharmaceutical Research Division, Takeda Pharmaceutical Company Ltd., Fujisawa, Kanagawa, Japan; Yong Loo Lin School of Medicine, National University of Singapore, SINGAPORE

## Abstract

Elucidating the bioactive compound modes of action is crucial for increasing success rates in drug development. For anticancer drugs, defining effective drug combinations that overcome resistance improves therapeutic efficacy. Herein, by using a biologically annotated compound library, we performed a large-scale combination screening with Stearoyl-CoA desaturase-1 (SCD1) inhibitor, T-3764518, which partially inhibits colorectal cancer cell proliferation. T-3764518 induced phosphorylation and activation of AMPK in HCT-116 cells, which led to blockade of downstream fatty acid synthesis and acceleration of autophagy. Attenuation of fatty acid synthesis by small molecules suppressed the growth inhibitory effect of T-3764518. In contrast, combination of T-3764518 with autophagy flux inhibitors synergistically inhibited cellular proliferation. Experiments using SCD1 knock-out cells validated the results obtained with T-3764518. The results of our study indicated that activation of autophagy serves as a survival signal when SCD1 is inhibited in HCT-116 cells. Furthermore, these findings suggest that combining SCD1 inhibitor with autophagy inhibitors is a promising anticancer therapy.

## Introduction

Cancer is still a major life-threatening disease despite significant progress in diagnostic technologies and medications [1]. Although many drug discovery studies have made great efforts to meet the need for new innovative cancer therapies, attrition rates during clinical trials remain high [2] because the lack of information regarding predictive biomarkers which reflect cancer vulnerability to drug candidates makes it difficult to enroll appropriate patients [3]. Therefore, detailed studies revealing a candidate compound’s mode(s) of action (MOA) are necessary to identify biomarkers that stratify patients, thereby increasing the success rate of clinical trials. In addition, it is also necessary to identify the appropriate combination partners for drug candidates, which more effectively address issues with tumor heterogeneity. Combination therapy is also typically more effective against the emergence of drug-resistant cancer cells than single drug therapies [4, 5]. Furthermore, most cancer cells contain mutations in driver genes, which are not always directly “druggable” [6]. Thus, the concept of synthetic lethality has received much attention because perturbation of two or more druggable targets would be equivalent to perturbation of a cancer driver gene. Recently, synthetic lethality has been shown with several drug combinations. For example, high sensitivity of BRCA mutants to PARP inhibitors is well-known in clinical settings [7]. Therefore, both MOA studies and combination partner screenings are necessary for successful cancer drug discovery and development. To achieve these goals, effective and straightforward technologies must be developed and implemented.

Functional genomics methods using gene silencing or editing technologies, such as small interfering RNA (siRNA)/short hairpin RNA (shRNA) [8, 9] or clustered regularly interspaced short palindromic repeats-Cas9 (CRISPR-Cas9) [10], are powerful tools for investigating MOAs and identifying synthetic lethal partners of small molecules because they are largely genome-wide approaches [11, 12]. However, functional genomics would not work well when an emerging phenotype requires intervention against all subtypes of a gene family. Furthermore, even if these genomic approaches lead to the discovery of partner genes or pathways, for clinical use, they need to be targeted by small molecule-based therapies.

Alternatively, combination therapy studies using small molecules are limited in their coverage of genes. However, it is easy to run a large-scale, high-throughput screening with small molecule libraries [13, 14], and the results might be clinically applicable. Recently, use of a focused compound library for phenotypic screening has been reported [15–18]. These libraries consist of compounds with known molecular targets; in other words, they are biologically annotated. After screening campaigns, the results can be used for target or pathway enrichment analysis and may lead to discovery of new connectivity [19].

Targeting cancer metabolism has opened new doors for innovative drug discovery [20], and drug candidates targeting this process have entered into clinical trials [21, 22]. Stearoyl-CoA desaturase-1 (SCD1) is a key molecule in fatty acid metabolism and has been recognized as a promising target for anticancer drugs [23]. SCD1 inhibitors, however, only show partial inhibition of HCT-116 colorectal cancer cell growth. Identification of combination partners and MOAs, therefore, could increase the efficacy of SCD1 inhibitors as anticancer drugs.

In this study, by using a biologically annotated compound library, we performed an unbiased, large-scale combination screening with SCD1 inhibitor, T-3764518, and unveiled the underlying mechanisms for resistance of HCT-116 cells against SCD1 inhibition. SCD1 knock-out (KO) cells generated with CRISPR-Cas9 technology were used to validate results obtained with small molecules. By using this simple and straightforward technology, we are able to detect effective combination partners in an unbiased manner, thereby increasing the efficacy of anticancer drugs.

## Materials and methods

### Cell culture

HCT-116 cells were purchased from American Type Culture Collection (ATCC; Manassas, VA, USA) and cultured in RPMI medium supplemented with 10% fetal bovine serum (Moregate, Brisbane, Australia), and 1× penicillin/streptomycin at 37°C and 5% CO_2_. A MycoAlert Mycoplasma Detection Kit (Lonza, Basel, Switzerland) was used to confirm that the cells were free from mycoplasma contamination.

### Chemicals

T-3764518 (SCD1 inhibitor) [24] and compound 7a [acetyl-CoA carboxylase (ACC) 1/2 dual inhibitor] [25] were synthesized by Takeda Pharmaceutical Company. AZD8055 [mammalian target of rapamycin (mTORC) inhibitor] and STA5326 (PIKfyve inhibitor) were purchased from Selleck (Houston, TX, USA). Vacuolin-1 (PIKfyve inhibitor) [26] was purchased from Merck Millipore (Darmstadt, Germany). GSK2194069 [fatty acid synthase (FASN) inhibitor] was purchased from Chemexpress Co., Ltd. (Shanghai, China). The Bax channel blocker (#2160) was purchased from Tocris (Bristol, UK), and hydroxychloroquine was purchased from Sigma-Aldrich (St. Louis, MO, USA). E64d and pepstatin A were purchased from Peptide Institute Inc. (Osaka, Japan).

### Antibodies

Anti-AMP-activated protein kinase (AMPK; #5831; 1:1000), anti-phospho-AMPK [Thr172] (#2535; 1:1000), anti-ACC (#3676; 1:1000), anti-phospho-ACC [Ser79] (#11818; 1:1000), anti-S6K (#2708; 1:1000), and anti-phopho-S6K [Thr389] (#9234; 1:1000) were purchased from Cell Signaling Technology (Danvers, MA, USA). Anti-SCD1 (ab39969; 1:500), and anti-actin (ab6276; 1:5000) were purchased from Abcam (Cambridge, MA, USA). Anti-microtubule-associated protein 1A/1B-light chain 3 (LC3; PM036; 1:2000) was purchased from MBL (Nagoya, Japan). Secondary anti-rabbit (#7074; 1:2000) and anti-mouse (715-035-151; 1:10000) antibodies were purchased from Cell Signaling Technology and Jackson ImmunoResearch (West Grove, PA, USA), respectively.

### Biologically annotated compound library

The biologically annotated compound library was composed of about 6400 small molecules, selected based on their biological diversity [27]. The library contained bioactive compounds with 50% inhibitory concentration (IC_50_) or half-maximal response values (EC_50_) less than 1 μM on diverse targets in the internal/external databases, as well as commercially available sets of known pharmacologically active compounds.

### Combination screening with biologically annotated compound library

HCT-116 cells were seeded at 5000 cells/well in 384-well black plates (Corning, Corning, NY, USA). Twenty-four hours after cell seeding, 3 μM of each test compound was added to cells with either 100 nM T-3764518 (combination condition) or with dimethylsulfoxide (DMSO) (single condition). After 72 h, cell viability was measured using a CellTiter-Glo^®^ Luminescent Cell Viability Assay (Promega, Madison, WI, USA) according to the manufacturer’s instructions with an EnVision Multilabel Reader (PerkinElmer, Waltham, MA, USA). For the single condition, wells containing no test compound and wells with no cells were used as 0% and 100% growth inhibition controls, respectively. For the combination condition, wells containing only T-3764518 and wells with no cells were used as 0% and 100% growth inhibition controls, respectively. Furthermore, we simplified the Bliss independence model to determine apparent combination effects. The calculated percent inhibition for each combination and single condition was used for x–y two-dimensional plotting. If the percent inhibition of a combination condition (y-axis) was larger than that of a single condition (x-axis), the test compound was determined to have a synergistic effect with T-3764518 (above y = x). Similarly, if a plot was on y = x, that compound was determined to have an additive/independent effect with T-3764518. If a plot was below y = x, that compound was determined to have an antagonistic effect with T-3764518. Moreover, for characterization of combination effects, each test compound and T-3764518 were serially diluted and added as a matrix. Data were then used for Bliss sum analysis and calculation of Bliss sum scores [28].

### Immunoblotting analysis

Cells seeded in 6-well plates and treated with compounds were washed with phosphate-buffered saline (PBS) twice to remove media. Cells were then incubated in lysis buffer (Cell Signaling Technology) supplemented with 1 mM phenylmethylsulfonyl fluoride on ice for 30 min. Lysates were centrifuged at 14,000 × g for 10 min at 4°C, and the supernatants were collected. Protein concentrations were determined by BCA protein assay (Thermo Fisher Scientific, Waltham, MA, USA). Lysates were boiled with SDS sample buffer (Bio-Rad, Hercules, CA, USA) containing 100 mM dithiothreitol (DTT) for 5 min. Samples were subjected to SDS-polyacrylamide gel electrophoresis (Wako Pure Chemical Co., Osaka, Japan), transferred to a Polyvinylidene Difluoride (PVDF) membrane with an iBlot apparatus (Thermo Fisher Scientific), and immunoblotted with indicated antibodies. Protein-antibody complexes were detected using enhanced chemiluminescence detection reagents (PerkinElmer).

### SCD1 KO by CRISPR-Cas9

HCT-116 cells were seeded at 2.5 × 10^5^ cells in 10-cm dishes and incubated in a CO_2_ incubator at 37°C. After 48 h, cells were transfected with plasmids encoding Cas9 nuclease (pCAGGS/Cas9), puromycin resistant genes (pEBMultipuro), and single guide RNAs targeting SCD1 using Lipofectamine LTX Reagent (Thermo Fisher Scientific). Guide RNA sequences used were 5′-GCAGAATGGAGGAGATAAGTTGG-3′ and 5′-GCCCCAAGGTTGAATATGTCTGG-3′. After 48 h, cells were trypsinized and replated in another 10-cm dish containing media supplemented with 0.5 μg/mL puromycin. After 10 days in culture, cells were trypsinized and seeded in 96-well plates at a density of 0.3–3 cells/well and cultured in medium without puromycin. SCD1 KO clones were selected by polymerase chain reaction. Genomic DNA prepared from clones obtained using SimplePrep reagent for DNA (Takara Bio) was used as a template, and the SCD1 genomic region was amplified with MightyAmp DNA Polymerase version 3 (Takara Bio). Primer sequences were 5′-ACCCTACCCTCAGTGAACTACG-3′ (forward) and 5′-GAAATGCCTGAGAAAAACCCCAA-3′ (reverse). Deletion of a part of the genomic region and expression of SCD1 protein were confirmed by genome sequencing and immunoblotting, respectively.

### LC3 dot formation assay

Cells seeded in 384-well black plates with clear bottoms (Cell Carrier Ultra; PerkinElmer) were treated with compounds for 24 h, then cells were fixed with 4% paraformaldehyde and permeabilized with 50 μg/mL digitonin (Wako Pure Chemical Co.) in PBS. After washing with PBS, cells were incubated with 10% goat serum (Thermo Fisher Scientific) for 1 h. After blocking, cells were incubated with anti-LC3 for 1 h, followed by incubation with an anti-rabbit secondary antibody conjugated to Alexa Fluor-488 (Thermo Fisher Scientific) and Hoechst-33258 for 1 h. After washing with PBS, images were captured by CV1000 (Yokogawa Electric Corp., Tokyo, Japan) using a 40× objective.

### GeneChip expression analysis

Cells were treated with DMSO or T-3764518 (100 nM) for 24 h, and RNA was prepared using an RNeasy Mini Kit (Qiagen, Valencia, CA, USA) according to the manufacturer’s instructions. GeneChip expression analysis was conducted at Takara Bio Inc. (Otsu, Shiga, Japan) using a Human Genome U133 Plus 2.0 Array. Gene expression data were deposited at Gene Expression Omnibus (Accession no. GSE98364).

### Statistical analysis

Growth inhibition data are expressed as the mean ± standard deviation of representative of more than two independent experiments. Each experiment contains at least four replicates. Statistical analysis was done with the unpaired t-test using GraphPad Prism software.

## Results

### Combination screening with T-3764518 using biologically annotated compound library

**Fig 1:**
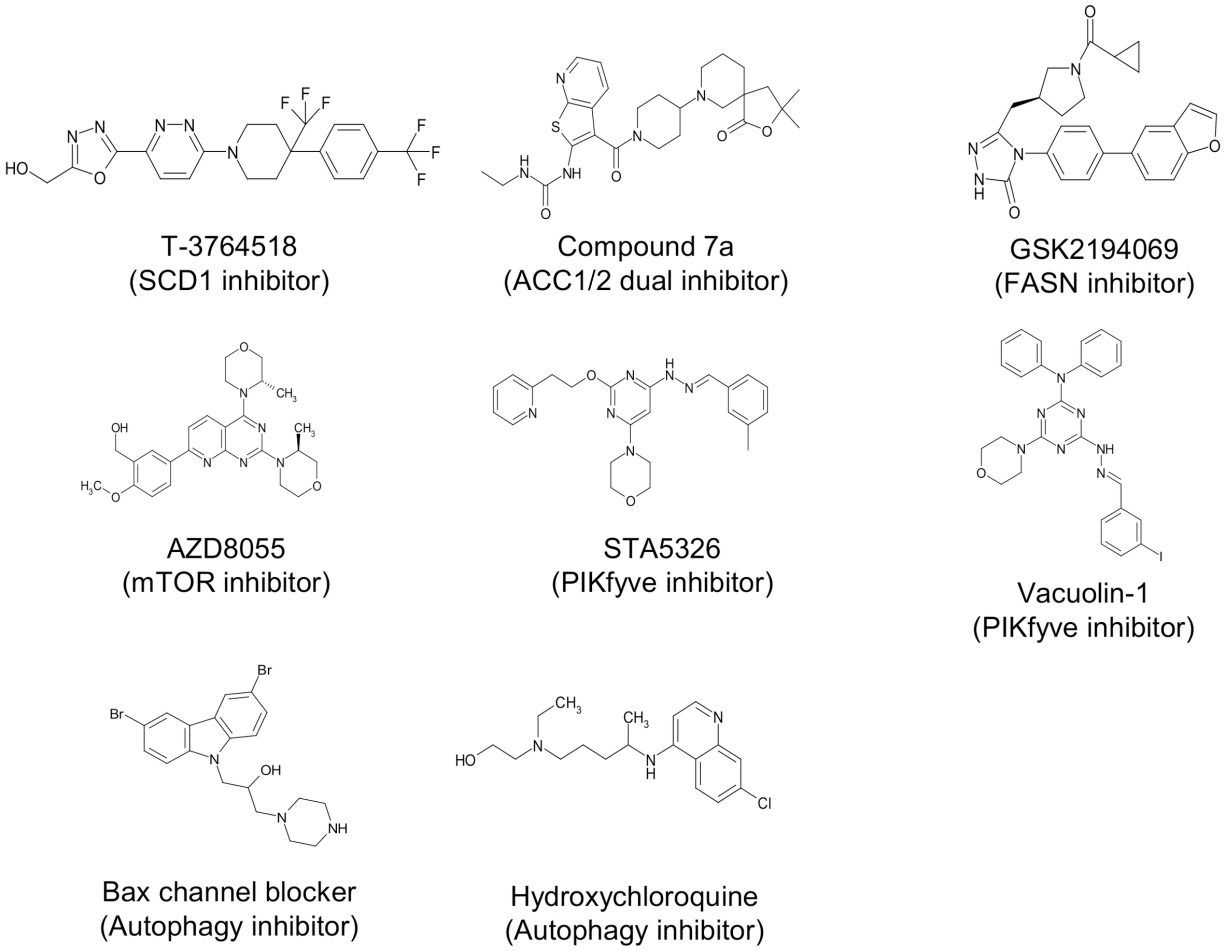
Chemical structure of compounds used. The reported MOA of each compound was also described.

**Fig 2:**
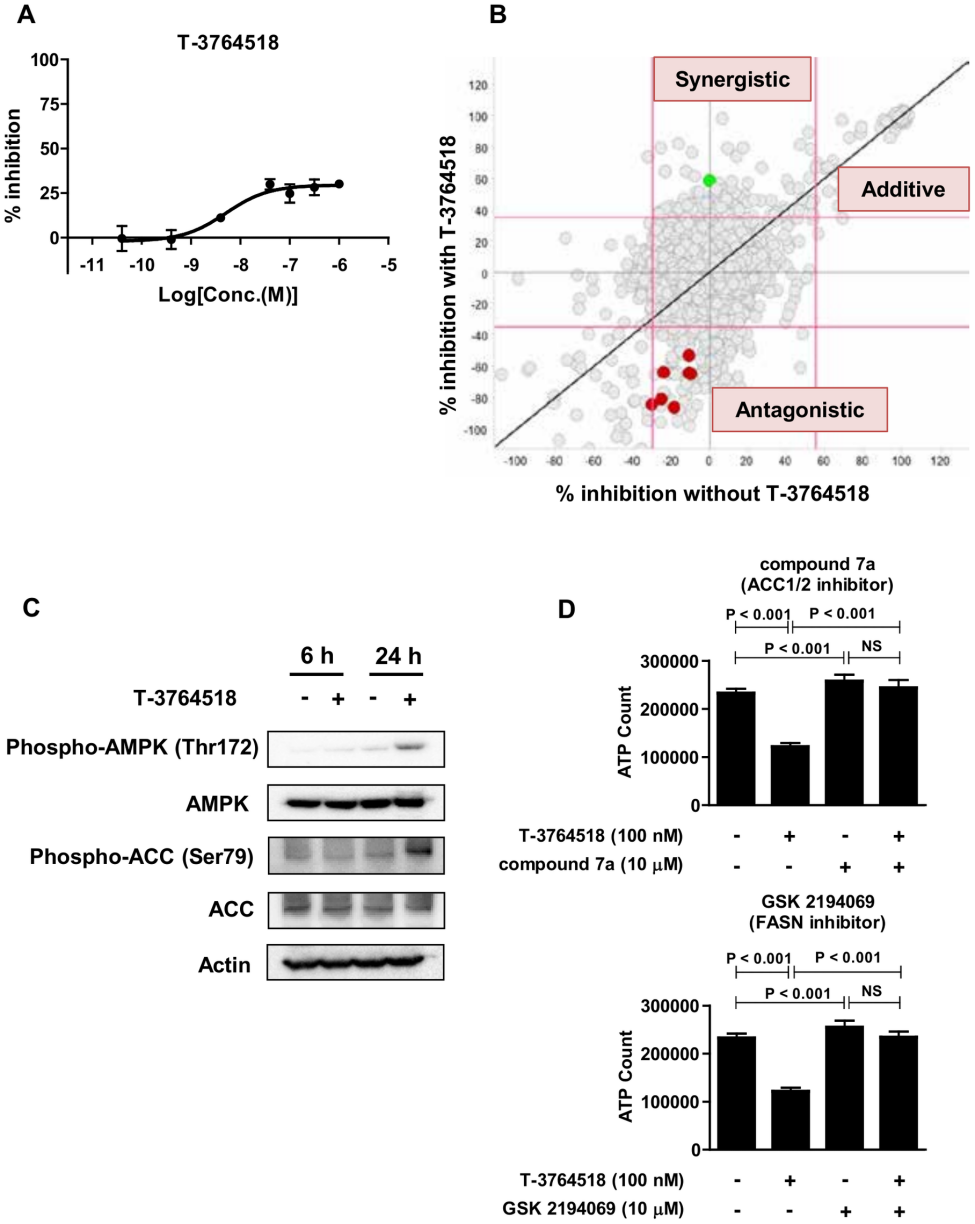
Inhibition of fatty acid synthesis cascade acted antagonistically to T-3764518. (A) Dose-response analysis of T-3764518 in HCT-116 cells treated with serial dilutions of T-3764518 for 72 h. Percent inhibition was normalized to wells treated with DMSO or no cells as 0% and 100% growth inhibition controls, respectively. Data was expressed as the mean ± standard deviation of representative of more than two independent experiments. Each experiment contains at least four replicates. (B) Summary of combination screening. Vertical and horizontal axes represent percent growth inhibition in the presence or absence of T-3764518, respectively. Red, ACC inhibitors; Green, Bax channel blocker. Based on the Bliss model, compounds were classified as synergistic, additive/independent, or antagonistic. (C) Western blot of HCT-116 cells treated with DMSO or T-3764518 (100 nM) for the indicated times; actin was used as a loading control. (D) Combination and single condition effects of compound 7a (10 μM) and GSK2194069 (10 μM) with T-3764518 (100 nM) on HCT-116 cells 72-h after treatment. Data was expressed as the mean ± standard deviation of representative of more than two independent experiments. Each experiment contains at least four replicates. NS, not significant by unpaired t-test.

In order to generate new drug candidates for cancer therapy, chemistry effort succeeded in identifying T-3764518 (Fig 1), which had an IC_50_ of 4.7 nM against recombinant SCD1 enzymatic activity [24]. However, T-3764518 only partially inhibited HCT-116 cell growth (Fig 2A), implying that its cellular potency is not strong enough to inhibit HCT-116 proliferation even though it achieves complete SCD1 inhibition at 10 μM, which is over 2000-fold higher than the enzymatic IC_50_ value. This result motivated us to elucidate the MOA by which T-3764518 inhibited cell growth and key pathways or molecules potentiating its growth inhibitory activity. To achieve this goal, we used a biologically annotated compound library to conduct a combination screening with T-3764518. The concentration of T-3764518 used for screenings was set at 100 nM since partial inhibition of HCT-116 cell growth was saturated at this concentration (Fig 2A). The concentration of library compounds used was set at 3 μM as it was considered that each compound would show their annotated activity at this concentration. Based on the screening results, tested library compounds were categorized as either synergistic, additive/independent, or antagonistic to the effects of T-3764518 via the Bliss independent model (Fig 2B). This model is widely accepted for comparing the combined and individual effects of different drugs [28, 29]. Detailed definitions of three categories are described in Materials and Methods.

### T-3764518 induced phosphorylation and activation of AMPK in HCT-116 cells

A series of ACC inhibitors, such as compound 7a (Fig 1), were found to be antagonistic to T-3764518 growth inhibition activity (Fig 2B, red plot). These results were unexpected since ACC1, one of the two isozymes of ACC, is upstream of SCD1 in the fatty acid synthesis cascade [30]. Thus ACC inhibitors were expected to show positive effects when combined with an SCD1 inhibitor. Previously, several reports suggested that SCD1 inhibition led to activation of AMPK through phosphorylation of Thr172 as a feedback mechanism [31–33]. AMPK is an upstream hub protein of ACC1/2 that negatively regulates each ACC isozyme by phosphorylating Ser79 and Ser222 residues, respectively. Therefore, we speculated that AMPK activation may cause resistance to the SCD1 inhibitor in our assay conditions and that further ACC inhibition would accelerate this resistance through the same cascade. Immunoblotting confirmed the existence of such feedback in HTC-116 cells (Fig 2C) as phospho-AMPK (Thr172) and phospho-ACC (Ser79) levels were increased after 24-h T-3764518 treatment. From these findings, we concluded that SCD1 inhibition triggered AMPK activation and following ACC inhibition in HCT-116 cells.

### Attenuated fatty acid synthesis is a resistant mechanism of HCT-116 cells against T-3764518

To examine whether further blockade of fatty acid synthesis was antagonistic to T-3764518 activity in HCT-116 cells, we examined inhibition of FASN, which is downstream of ACC in the fatty acid synthesis cascade. As shown in Fig 2D and Panel A in S1 Fig, GSK2194069 (FASN inhibitor) and compound 7a (ACC1/2 inhibitor) suppressed the growth inhibition activity of T-3764518 in a dose-dependent manner. Combination matrix experiments were performed to evaluate each combinatorial effect in detail by calculating Bliss sum [34]. Bliss sum values of T-3764518/GSK2194069 and T-3764518/compound 7a were −550 and −569, respectively (Panel B in S1 Fig), indicating that both were antagonistic to T-3764518 activity. Furthermore, we assessed whether siRNA-mediated knockdown of AMPK increased the sensitivity of HCT116 cells to T-3764518. Treatment of HCT116 cells with siRNAs targeting AMPK (PRKAA1 and PRKAA2) resulted in the enhancement of the cell growth inhibitory effect of T-3764518 (Panel C in S1 Fig). Collectively, these results revealed that the feedback activation of AMPK caused by SCD1 inhibition and resultant attenuation of downstream fatty acid synthesis cascade was an underlying mechanism of HCT-116 resistance to T-3764518 treatment.

### T-3764518 induces autophagy in HCT-116 cells and combination treatment with autophagy inhibitors increases sensitivity to T-3764518

**Fig 3:**
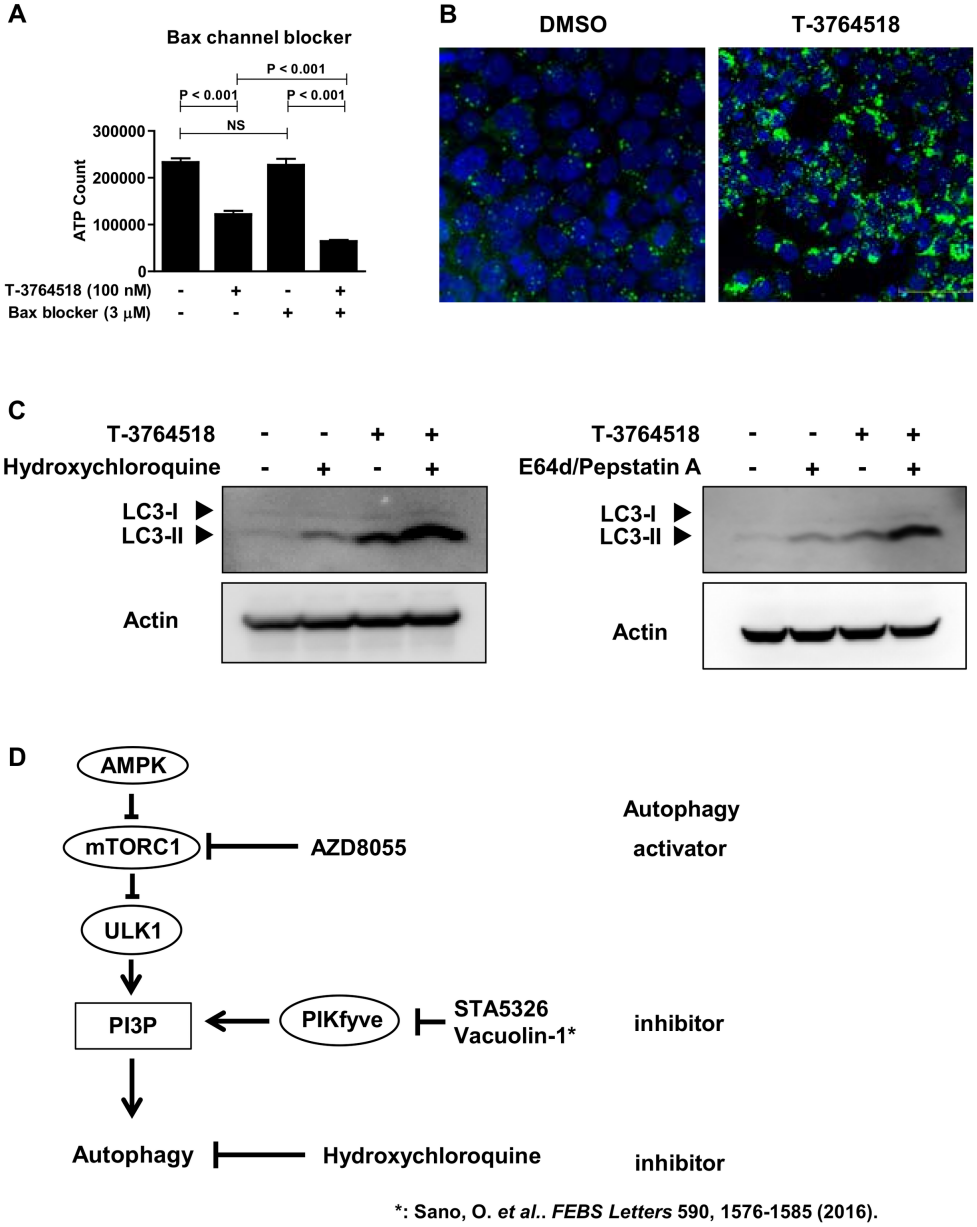
T-3764518 treatment induced autophagy in HCT-116 cells. (A) Effect of 72-h Bax channel blocker (3 μM) treatment of HCT-116 cells with or without T-3764518 (100 nM). Data was expressed as the mean ± standard deviation of representative of more than two independent experiments. Each experiment contains at least four replicates. NS, not significant by unpaired t-test. (B) Representative images of LC3 dot formation in HCT-116 cells treated with T-3764518 (100 nM) for 24 h and then fixed and stained with Hoechst-33258 (blue) and anti-LC3 (green). (C) Western blot of HCT-116 cells treated with hydroxychloroquine (10 μM) or E64d (10 μg/ml) and pepstatin A (10 μg/ml) with or without T-3764518 (100 nM) for 24 h; actin was used as a loading control. (D) Autophagy cascade and sites of compound action used for further study in Fig 4 are shown.

**Fig 4:**
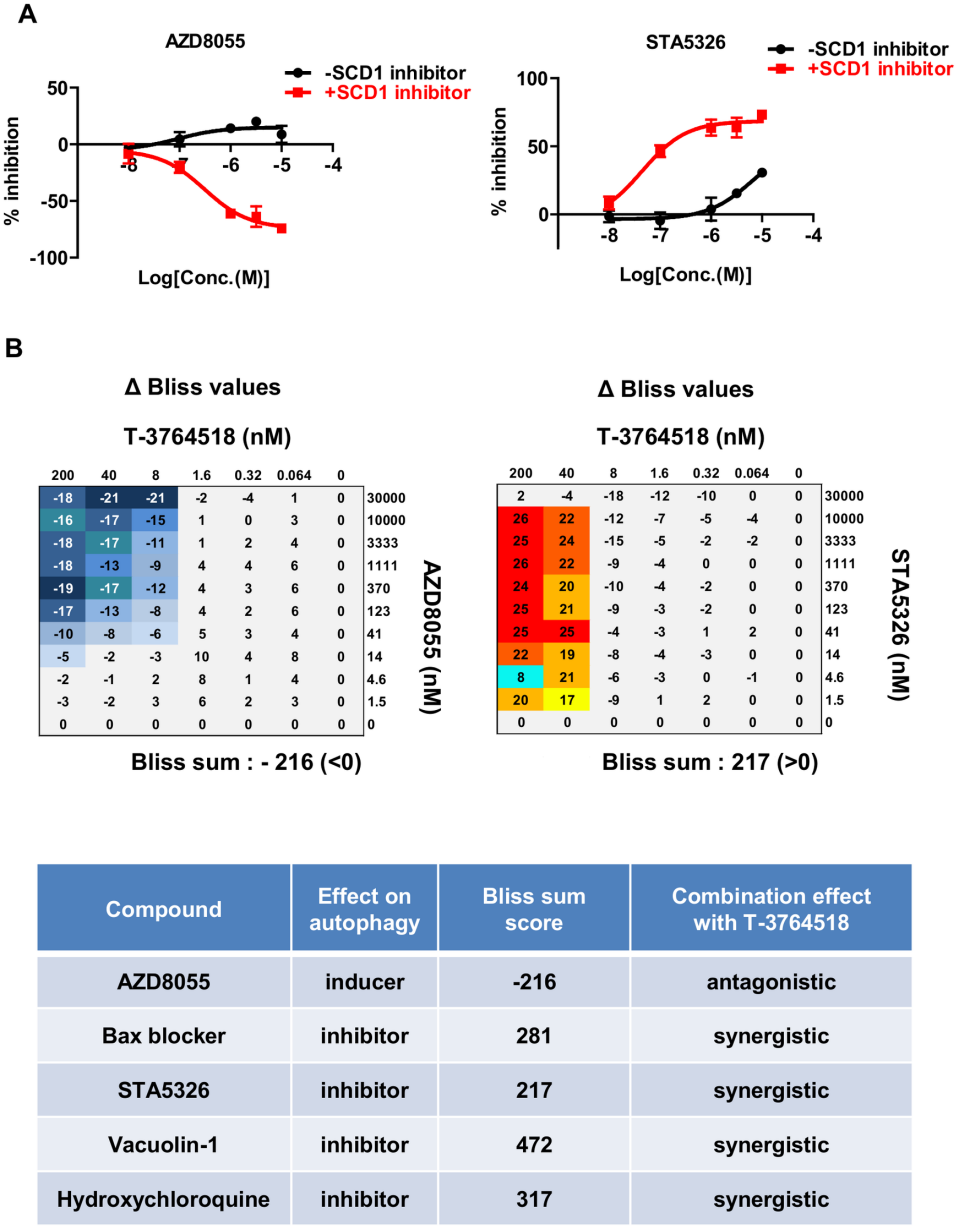
Combinatorial effects of autophagy inducers and inhibitors with T-3764518 in HCT-116 cells. (A) Combinatorial effects of serially diluted AZD8055 or STA5326 with T-3764518 (100 nM) in HCT-116 cells after 72 h. Data was expressed as the mean ± standard deviation of representative of more than two independent experiments. Each experiment contains at least four replicates. (B) Drug matrix heatmap illustrating ΔBliss values. HCT-116 cells were treated with AZD8055 or STA5326 alone or in combination with T-3764518 at the indicated concentrations. Bliss sum scores >0 indicate a synergistic effect; Bliss sum scores <0 indicate an antagonistic effect.

Among synergistic compounds selected from screening, Bax channel blocker was subjected to further investigation because it was a hit compound from the autophagy inhibitor screening campaign that we carried out previously [26] (Fig 2B, green plot). Bax channel blocker (3 μM) potentiated the growth inhibitory activity of T-3764518 in HCT-116 cells (Fig 3A and Panel A in S2 Fig). In addition, as shown above, T-3764518 induced AMPK activation (Fig 2C), which was a key positive regulator of autophagy [35]. Therefore, we hypothesized that autophagy inhibiting compounds would be synergistic with T-3764518 since HCT-116 cells would upregulate autophagy for survival by activating AMPK. To test this hypothesis, we first examined whether autophagy was accelerated by T-3764518 in HCT-116 cells using LC3 dot formation assays. T-3764518 treatment increased LC3 dot formation in HCT-116 cells compared to DMSO-treated cells (Fig 3B). Similarly, immunoblotting revealed that the lipidated form of LC3, LC3-II, was increased by T-3764518 treatment (Fig 3C). Furthermore, pharmacological inhibition of autophagy using hydroxychloroquine or E64d/pepstatin A, that block degradation of autolysosome content [36], enhanced the accumulation of LC3-II in HCT116 cells treated with T-3764518 (Fig 3C), indicating that T-3764518 treatment led to the acceleration of autophagic flux. We then evaluated the combinatorial effects of T-3764518/autophagy modulators (AZD8055, STA5326, vacuolin-1, or hydroxychloroquine) on HCT-116 cell growth (Fig 3D). While AZD8055 was antagonistic to T-3764518 activity (Fig 4A), STA5326, vacuolin-1, and hydroxychloroquine all exhibited synergistic effects (Fig 4A and Panel A in S2 Fig). As shown in Fig 4B, the Bliss sum value for AZD8055 was less than 0 (antagonistic), whereas Bliss sum values for the autophagy inhibitors were all greater than 0 (synergistic; Fig 4B and Panel B in S2 Fig). Since a metabolic assay, such as an ATP measurement, may not reflect accurate cytotoxicity of compounds under some experimental conditions, we confirmed the similar combination effects using cellular DNA contents as a cell proliferation marker instead of cellular ATP contents (Panel C in S2 Fig). Collectively, these results were consistent with the hypothesis that autophagy upregulation contributed to T-3764518 resistance in HCT-116 cells. Taken together, we concluded that activation of AMPK and following induction of autophagy was an underlying mechanism of HCT-116 cell resistance to T-3764518 treatment. To verify that the combinatorial effects of T-3764518 was not cell-type specific, other colorectal cancer cells, HCT-15, HT-29, and SW620 cells, were treated with compound 7a or Bax channel blocker alone or in combination with T-3764518. We confirmed antagonistic and synergistic effects of each compound to T-3764518 similar to those observed in HCT116 cells (Panel D in S2 Fig). These findings suggested that attenuation of fatty acid synthesis and induction of autophagy served as an universal resistant mechanism against SCD1 ablation at least in some colorectal cancer cells.

### Validation of the results obtained by T-3764518 using SCD1-KO cells

**Fig 5:**
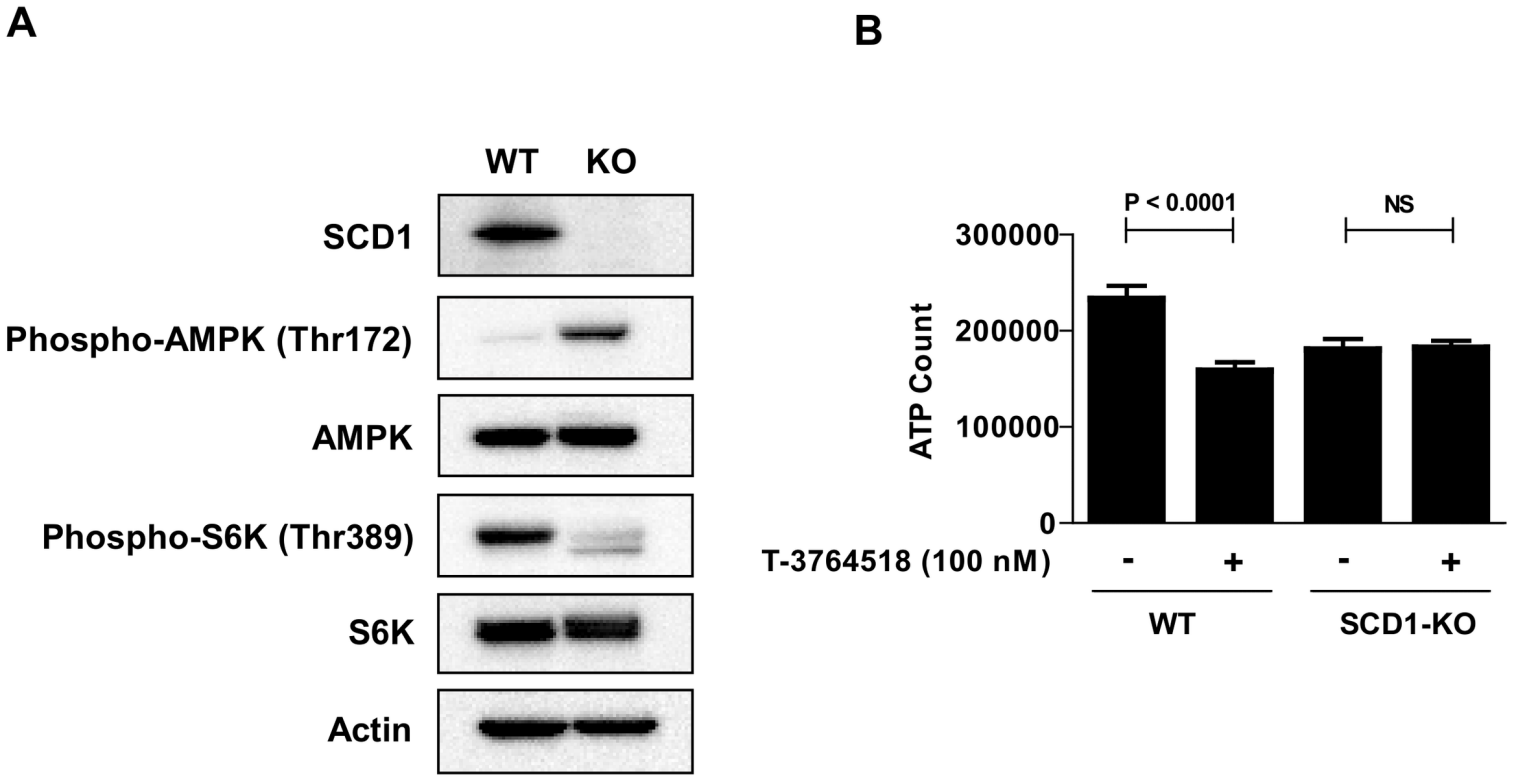
Validation of the results obtained by T-3764518 with SCD1-KO cells. (A) Western blot analysis of SCD1-KO cell lysates; actin was used as a loading control. (B) Growth inhibitory effect of T-3764518 (100 nM) on SCD1-KO and SCD1-WT cells after 72 h of treatment. Data was expressed as the mean ± standard deviation of representative of more than two independent experiments. Each experiment contains at least four replicates. NS, not significant by unpaired t-test.

SCD1-KO HCT-116 cells were generated with CRISPR-Cas9 technology to strengthen the hypothesis derived from the results obtained by T-3764518. The absence of SCD1 protein was confirmed by western blotting using an anti-SCD1 antibody (Fig 5A). Western blot analysis of SCD1-KO cell lysates also showed that phospho-AMPK (Thr172) levels were elevated compared to SCD1-wild-type (WT) cells (Fig 5A), indicating that the feedback occurred in SCD1-KO cells as observed in SCD1-WT cells treated with T-3764518. Phosphorylation of S6K, a downstream effector of mTOR, was decreased in SCD1-KO cells, suggesting downregulation of mTOR via AMPK activation. Moreover, these cells were no longer sensitive to T-3764518 (Fig 5B). LC3 dot formation assay showed upregulation of autophagy in SCD1-KO cells relative to SCD1-WT cells (Panel A in S3 Fig). These results suggested that AMPK activation and mTOR inhibition lead to upregulation of autophagy in the absence of SCD1. Furthermore, we found that SCD1-KO cells were more sensitive to autophagy inhibitors than SCD1-WT cells (Panel B in S3 Fig). Collectively, these results indicated that autophagy served as a survival signal in SCD1-KO cells. Next, we performed GeneChip expression analysis using SCD1-WT and SCD1-KO cells treated with DMSO or T-3764518 (S1 Table). Fatty acid synthesis- and/or autophagy-related genes were not found among commonly downregulated genes in both SCD1-WT cells treated with T-3764518 and SCD1-KO cells treated with DMSO. Conversely, we observed that expression of *SCD* and *FASN* mRNA was upregulated over 2-fold in both SCD1-WT cells treated with T-3764518 and SCD1-KO cells treated with DMSO relative to SCD1-WT cells treated with DMSO (S4 Fig). On the other hand, as aforementioned, fatty acid synthesis was functionally attenuated as indicated by increased phospho-ACC levels (Fig 2C). These results suggested that cells compensated for SCD1 perturbation by increasing levels of *SCD* and *FASN* mRNA to maintain homeostasis. Meanwhile, we observed that *MAP1LC3B* mRNA levels were upregulated in both SCD1-WT cells treated with T-3764518 and SCD1-KO cells, consistent with increased LC3 dot formation. Thus, we confirmed at the transcriptional level that SCD1 perturbation affected fatty acid synthesis and autophagy in HCT-116 cells.

## Discussion

**Fig 6:**
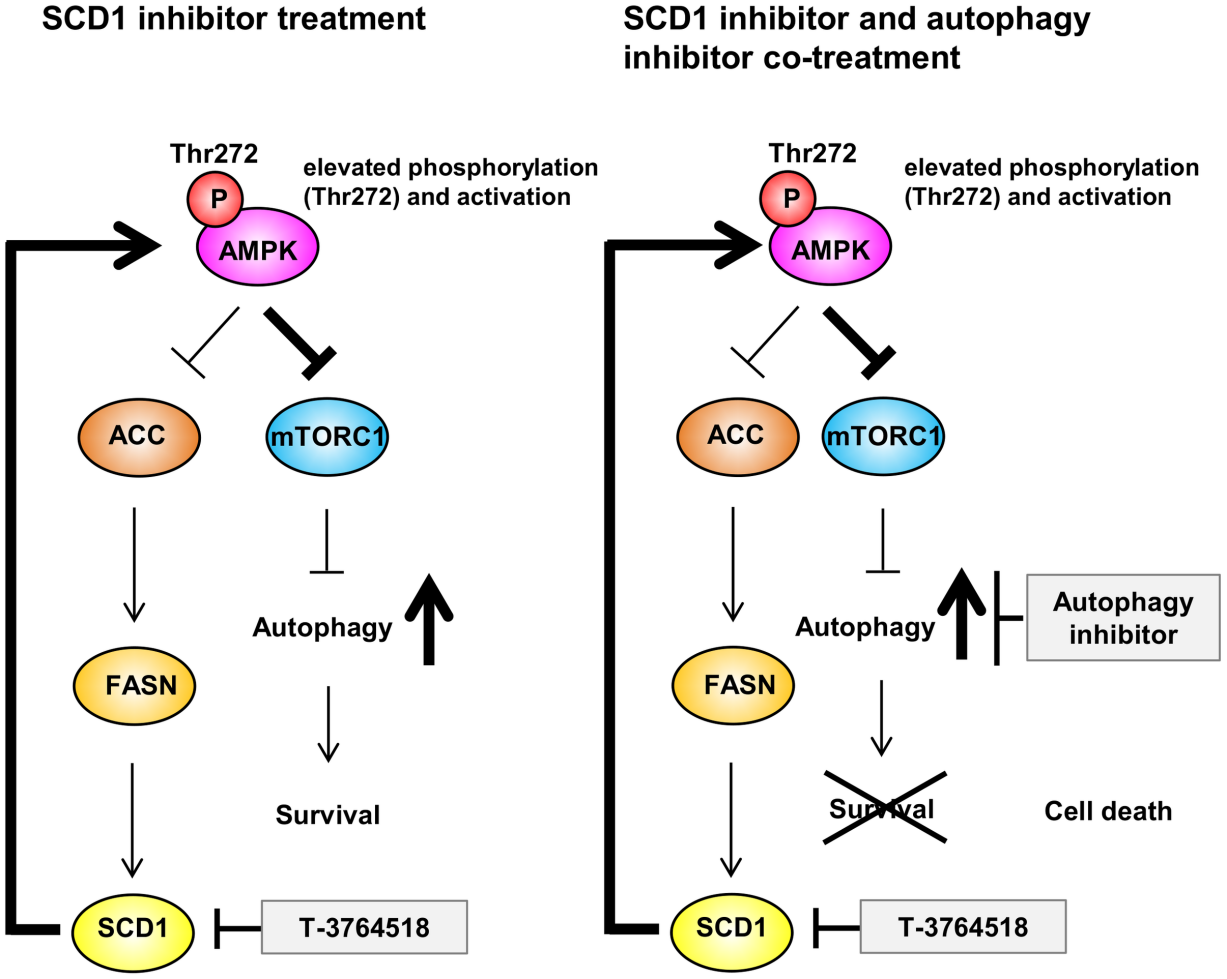
Growth inhibition mode of action (MOA) summary for T-3764518. Autophagy activation functions as a survival signal in cells treated with T-3764518. Dual inhibition of SCD1 and autophagy may be an effective strategy to combat cancer.

Despite the significant progress that has been made in diagnostic technologies and medications, cancer is still a major cause of death worldwide. One reason is our poor understanding of MOAs and effective combination partners for drug candidates. Although functional genomics tools, such as CRISPR and shRNA, are powerful approaches for elucidation of synthetic lethal partner genes, identified candidate genes are often undruggable [11]. Thus, herein, we used a biologically annotated compound library to find pathways which modulate the sensitivity of HCT-116 cells to the SCD1 inhibitor, T-3764518. We revealed multiple mechanisms by which HCT-116 colorectal cancer cells become resistant to T-3764518-induced SCD1 inhibition using both small molecule and SCD1-KO cell studies (Fig 6). SCD1 inhibition was found to activate AMPK through a feedback system in HCT-116 cells, leading to the blockade of downstream fatty acid synthesis cascade. Attenuation of fatty acid synthesis probably served as a mechanism of resistance to SCD1 inhibition since further blockade of this synthetic cascade by small molecules rendered cells more resistant to T-3764518. Although the mechanisms underlying T-3764518-triggered cell death are not fully understood, we speculate that accumulation of saturated fatty acids (SFA; i.e., SCD1 substrates [37]) in HCT-116 cells causes cell death triggered by SCD1 inhibition. When SCD1 is inhibited, HCT-116 cells would reduce their levels of SFA by attenuating upstream fatty acid synthetic protein activity in order to survive. Hess *et al*. have reported that co-treatment of lung cancer cells with SCD1 inhibitor and ACC inhibitor did not potentiate the growth inhibitory effect of these compounds [38]. Contrary to expectations, in our models, pharmacological inhibition of ACC or FASN fully rescued the growth inhibitory effect of T-3764518 as shown in Fig 2D. Based on these results, we speculated that accumulation of SFA is deleterious in cells when SCD1 was inhibited by T-3764518. This hypothesis is in agreement with the study by Scaglia *et al*. in which they suggested that lung cancer cells downregulated SFA synthesis to prevent harmful effects of SFA accumulation when SCD1 was inhibited by CVT-11127 [33]. Our results suggest that the similar preventive mechanism against growth inhibition triggered by SCD1 inhibition also exists in colorectal cancer cells. Recently, Nishizawa *et al*. have also reported that exogenous SFA enhanced the growth inhibitory effect of T-3764518 in HCT-116 cells [39]. Collectively, although we did not rule out the possibility that monounsaturated fatty acids (products of SCD1) contributes to a part of resistance of T-3764518-treated cells as we used media containing FBS without charcoal treatment in our experiments, our results suggested that attenuation of SFA synthesis would be a primary resistant mechanism in T-3764518-treated HCT116 cells. Alternatively, activated AMPK also blocked mTOR activity, leading to acceleration of autophagy, which served as a survival signal in HCT-116 cells (Fig 6, left). When cells were treated with a combination of SCD1 and autophagy inhibitors, activation of autophagy as a survival signal was suppressed, leading to cancer cell death (Fig 6, right). The roles of SCD1 in autophagy remain controversial. Our observations are in agreement with some previous studies in which SCD1 inhibition accelerated autophagy [40, 41]. Huang *et al*. and Tan *et al*. reported that CAY-10566, a SCD1 inhibitor, induced autophagy in hepatocellular carcinoma cell lines and in TSC2^-/-^ MEF cells, respectively. On the other hand, Ogasawara *et al*. reported that another SCD1 inhibitor, 28c, inhibited autophagy in MEF cells [42]. This apparent discrepancy may be due to the differences in experimental conditions among studies, such as cells and incubation time with compounds. Ogasawara *et al*. proved that starvation-induced autophagy was blocked by short term (2 h) SCD1 inhibitor treatment [42]. Alternatively, we and the other two groups investigated the effects on constitutive, not starvation-induced, autophagic flux under long term (24 h) SCD1 inhibition [40, 41]. In summary, our results demonstrated that HCT-116 cells escaped death caused by SCD1 inhibition by at least two mechanisms: 1) feedback attenuation of fatty acid synthesis and 2) acceleration of autophagy.

Small molecules often possess off-targets, which makes it difficult to identify key molecules relevant to the phenotype triggered by the compound. To overcome this drawback, we utilized SCD1-KO cells generated using CRISPR-Cas9 technology. We observed identical phenotypes in both SCD1-WT cells treated with T-3764518 and SCD1-KO cells. Therefore, our results suggest that the phenotypes obtained by T-3764518 in HCT-116 cells were directly due to SCD1 inhibition. We also performed GeneChip analysis using SCD1-WT cells treated with T-3764518 and SCD1-KO cells. Among the genes commonly upregulated in both cell types, fatty acid synthesis-related (*FASN* and *SCD*) and autophagy-related (*MAP1LC3B*) genes were identified. Thus, we confirmed that SCD1 inhibition modulates fatty acid synthesis and autophagy at the transcriptome level. It is often laborious to analyze GeneChip data because many genes are upregulated or downregulated in response to stimulation. By comparing gene expression profiles in SCD-WT cells treated with T-3764518 and SCD1-KO cells, we were able to efficiently identify meaningful changes in gene expression related to SCD1 inhibition. Therefore, this approach, which combines chemical and genetic analytical tools, is a powerful strategy to study MOAs of small molecules and strengthens hypotheses derived from small molecule-based approaches.

From a clinical viewpoint, our observations suggest that combined SCD1 inhibitor/autophagy inhibitor treatment could be an effective anticancer therapy. Clinical studies using an mTORC inhibitor (temsirolimus) and an autophagy inhibitor (hydroxychloroquine) in combination have been undertaken and showed positive results for advanced solid tumors and melanoma [43]. In line with our findings, this result strongly indicates that autophagy activation may play an important role in resistance of cancer cells against anticancer therapies. Von Roemeling *et al*. reported several effective combinatorial treatments using A939572, a SCD1 inhibitor [44, 45]. They found that combination of A939572 with temsirolimus synergistically enhanced tumor cell death in clear cell renal cell carcinoma cell lines. This result was not consistent with the combinatorial effect we have observed using T-3764518 and AZD8055. Temsirolimus has been previously found to lower SCD1 expression levels in cancer cells [46]. Thus, the synergistic effect of temsirolimus with A939572 may be due to additional inhibition of SCD1 at a transcriptional level by temsirolimus.

In conclusion, by using both chemical and genetic tools, we have unveiled the underlying mechanisms for resistance of colorectal cancer cells against SCD1 inhibition. Feedback activation of AMPK-mediated autophagy acceleration serves as a key resistance mechanism in colorectal cancer cells. Our methodology is simple, straightforward, and able to detect novel, effective combination partners, which will enable us to improve the success rates of anticancer drug development.

## Supporting information

S1 Fig Combinatorial effect of ACC and FASN inhibitors with T-3764518 in HCT-116 cells. (A) Effects of serially diluted compound 7a (ACC inhibitor) or GSK2194069 (FASN inhibitor) with or without T-3764518 (100 nM) on HCT116 cells after 72 h of treatment. Data was expressed as the mean ± standard deviation of representative of more than two independent experiments. Each experiment contains at least four replicates. (B) Drug matrix heatmap illustrating ΔBliss values for HCT-116 cells treated with T-3764518 and compound 7a or GSK2194069 as single agents and in combination across a range of indicated concentrations. Cell proliferation was evaluated using a cellular ATP contents. A Bliss sum <0 indicates an antagonistic effect. (C) Effects of siRNAs targeting AMPK (PRKAA1 and PRKAA2) with or without T-3764518 on HCT116 cells after 72 h of treatment. Data was expressed as means ± SD (*n* = 4). Knockdown efficiencies were evaluated using Taqman qPCR assay. Data ware normalized to ACTB and calculated using the delta cycle threshold method.
(PDF)

S2 Fig Combinatorial effects of Bax channel blocker and vacuolin-1 with T-3764518 in HCT-116 cells. (A) Effects of serially diluted Bax channel blocker or vacuolin-1 with or without T-3764518 (100 nM) in HCT116 cells after 72 h of treatment. Data was expressed as the mean ± standard deviation of representative of more than two independent experiments. Each experiment contains at least four replicates. (B) Drug matrix heatmap illustrating ΔBliss values for HCT-116 cells treated with T-3764518 and Bax channel blocker, vacuolin-1, or hydroxychloroquine as single agents or in combination across a range of indicated concentrations. A Bliss sum >0 indicates a synergistic effect. (C) Drug matrix heatmap illustrating ΔBliss values for HCT-116 cells treated with combination of T-3764518 and each compound measured by cellular DNA contents as an indicator of cell proliferation. (D) Drug matrix heatmap illustrating ΔBliss values for other colorectal cancer cell lines, HCT-15, HT-29, and SW620 cells, treated with T-3764518 and each compound.
(PDF)

S3 Fig SCD1-WT and SCD1-KO cellular proliferation with autophagy inhibitor treatment. (A) Representative images of LC3 dot formation in SCD1-KO cells treated with T-3764518 (100 nM) for 24 h, and then fixed and stained with Hoechst-33258 (blue) and anti-LC3 (green). (B) Dose-response analysis of SCD1-WT and SCD1-KO cells treated with serial dilutions of Bax channel blocker and STA5326 for 72 h. Percent inhibition was normalized to wells treated with DMSO or no cells as 0% and 100% growth inhibition controls, respectively. Data was expressed as the mean ± standard deviation of representative of more than two independent experiments. Each experiment contains at least four replicates.
(PDF)

S4 Fig Fold-increase in *SCD*, *FASN*, and *MAP1LC3B* expression in HCT-116 cells. HCT-116 cells were treated with DMSO or T-3764518 for 24 h, and gene expression levels were analyzed via Human Genome U133 Plus 2.0 Array. Fold-increases for each gene in SCD1-WT cells treated with T-3764518 and SCD1-KO cells treated with DMSO relative to SCD1-WT cells treated with DMSO are shown.
(PDF)

S1 Text Materials and methods for supporting information. (DOCX)

S1 Table Signal intensity from GeneChip analysis data. (XLSX)
