# Supplementary figures and images for: Feedback activation of AMPK-mediated autophagy acceleration is a key resistance mechanism against SCD1 inhibitor-induced cell growth inhibition
Source: PLoS One. 2017 Jul 13;12(7):e0181243. doi: 10.1371/journal.pone.0181243 (PMC5509324; doi:10.1371/journal.pone.0181243)

**A**

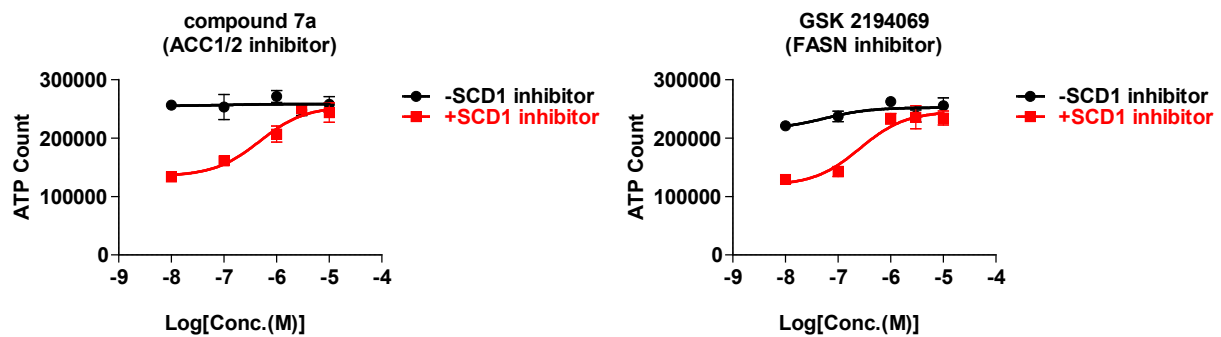

**B**

**Δ Bliss values**

**T-3764518 (nM)**

| 200 | 40  | 8   | 1.6 | 0.32 | 0.064 | 0 |       |
|-----|-----|-----|-----|------|-------|---|-------|
| -41 | -34 | -20 | -2  | -5   | -5    | 0 | 30000 |
| -36 | -31 | -19 | 1   | -3   | -3    | 0 | 10000 |
| -38 | -30 | -19 | -1  | -2   | -4    | 0 | 3333  |
| -31 | -26 | -14 | 4   | 4    | 4     | 0 | 1111  |
| -31 | -24 | -15 | 6   | -2   | 5     | 0 | 370   |
| -26 | -18 | -15 | 3   | -3   | -2    | 0 | 123   |
| -16 | -7  | -7  | -2  | -4   | 3     | 0 | 41    |
| -8  | -2  | -2  | 3   | 3    | 2     | 0 | 14    |
| -11 | -6  | -5  | -2  | -3   | -1    | 0 | 4.6   |
| -9  | -7  | -6  | -3  | -6   | -3    | 0 | 1.5   |
| 0   | 0   | 0   | 0   | 0    | 0     | 0 | 0     |

**compound 7a (nM)**

**Bliss sum : - 569 (<0)**

**Δ Bliss values**

**T-3764518 (nM)**

| 200 | 40  | 8   | 1.6 | 0.32 | 0.064 | 0 |       |
|-----|-----|-----|-----|------|-------|---|-------|
| -34 | -38 | -29 | -6  | -8   | -8    | 0 | 30000 |
| -30 | -26 | -19 | 2   | -3   | -3    | 0 | 10000 |
| -25 | -19 | -15 | 4   | 1    | -1    | 0 | 3333  |
| -30 | -26 | -15 | 6   | 3    | -1    | 0 | 1111  |
| -28 | -21 | -16 | 4   | 1    | 1     | 0 | 370   |
| -28 | -21 | -16 | 5   | 7    | 3     | 0 | 123   |
| -28 | -22 | -20 | -3  | -3   | -5    | 0 | 41    |
| -20 | -20 | -16 | 5   | 3    | 3     | 0 | 14    |
| -5  | -7  | -12 | 5   | 4    | 0     | 0 | 4.6   |
| 2   | 4   | 2   | 7   | 4    | 1     | 0 | 1.5   |
| 0   | 0   | 0   | 0   | 0    | 0     | 0 | 0     |

**GSK 2194069 (nM)**

**Bliss sum : - 550 (<0)**

C

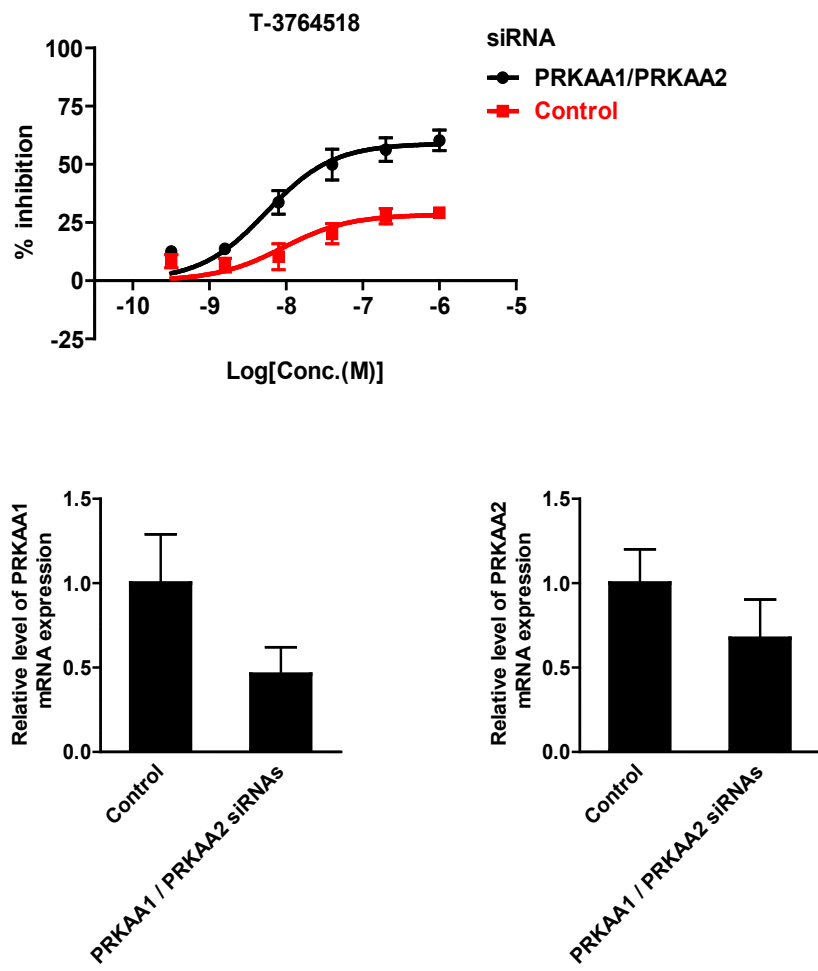

Supplement: S1 Fig — (A) Effects of serially diluted compound 7a (ACC inhibitor) or GSK2194069 (FASN inhibitor) with or without T-3764518 (100 nM) on HCT116 cells after 72 h of treatment. Data was expressed as the mean ± standard deviation of representative of more than two independent experiments. Each experiment contains at least four replicates. (B) Drug matrix heatmap illustrating ΔBliss values for HCT-116 cells treated with T-3764518 and compound 7a or GSK2194069 as single agents and in combination across a range of indicated concentrations. Cell proliferation was evaluated using a cellular ATP contents. A Bliss sum <0 indicates an antagonistic effect. (C) Effects of siRNAs targeting AMPK (PRKAA1 and PRKAA2) with or without T-3764518 on HCT116 cells after 72 h of treatment. Data was expressed as means ± SD (n = 4). Knockdown efficiencies were evaluated using Taqman qPCR assay. Data ware normalized to ACTB and calculated using the delta cycle threshold method. (PDF) [file pone.0181243.s001.pdf]

**A**

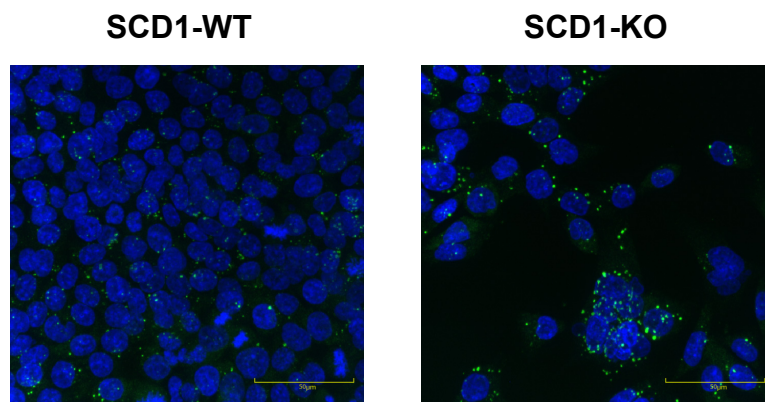

**B**

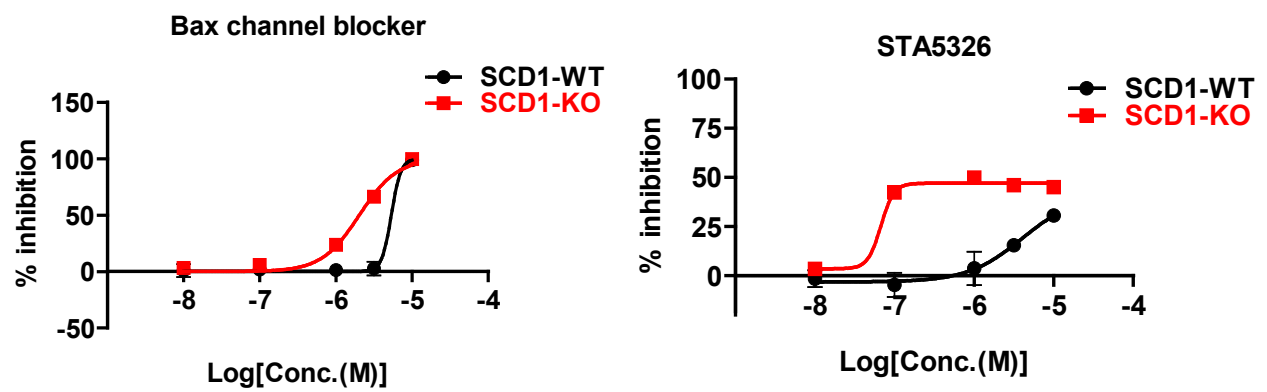

Supplement: S3 Fig — (A) Representative images of LC3 dot formation in SCD1-KO cells treated with T-3764518 (100 nM) for 24 h, and then fixed and stained with Hoechst-33258 (blue) and anti-LC3 (green). (B) Dose-response analysis of SCD1-WT and SCD1-KO cells treated with serial dilutions of Bax channel blocker and STA5326 for 72 h. Percent inhibition was normalized to wells treated with DMSO or no cells as 0% and 100% growth inhibition controls, respectively. Data was expressed as the mean ± standard deviation of representative of more than two independent experiments. Each experiment contains at least four replicates. (PDF) [file pone.0181243.s003.pdf]
