# Supplementary material for: Feedback activation of AMPK-mediated autophagy acceleration is a key resistance mechanism against SCD1 inhibitor-induced cell growth inhibition
Source: PLoS One. 2017 Jul 13;12(7):e0181243. doi: 10.1371/journal.pone.0181243 (PMC5509324; doi:10.1371/journal.pone.0181243)
Supplement: S2 Fig — (A) Effects of serially diluted Bax channel blocker or vacuolin-1 with or without T-3764518 (100 nM) in HCT116 cells after 72 h of treatment. Data was expressed as the mean ± standard deviation of representative of more than two independent experiments. Each experiment contains at least four replicates. (B) Drug matrix heatmap illustrating ΔBliss values for HCT-116 cells treated with T-3764518 and Bax channel blocker, vacuolin-1, or hydroxychloroquine as single agents or in combination across a range of indicated concentrations. A Bliss sum >0 indicates a synergistic effect. (C) Drug matrix heatmap illustrating ΔBliss values for HCT-116 cells treated with combination of T-3764518 and each compound measured by cellular DNA contents as an indicator of cell proliferation. (D) Drug matrix heatmap illustrating ΔBliss values for other colorectal cancer cell lines, HCT-15, HT-29, and SW620 cells, treated with T-3764518 and each compound. (PDF) [file pone.0181243.s002.pdf]

A

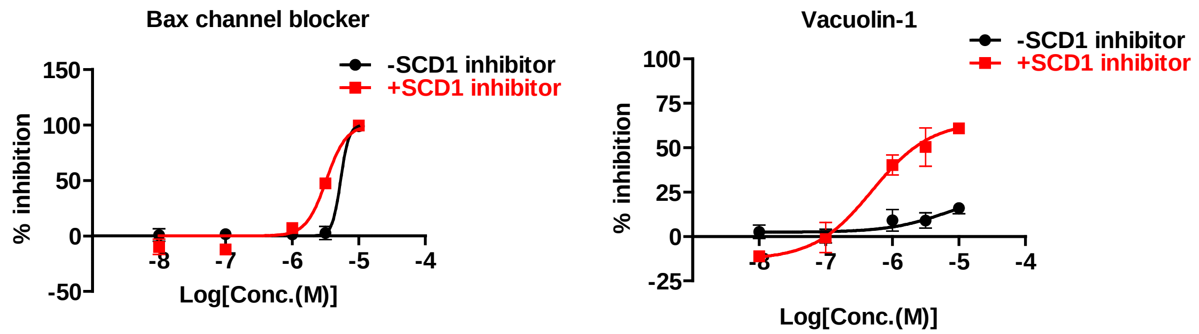

B

Δ Bliss values

T-3764518 (nM)

200	40	8	1.6	0.32	0.064	0	Bax channel blocker (nM)
0	0	0	0	0	0	0	30000
0	0	0	0	0	0	0	10000
53	57	61	-4	-5	-3	0	3333
39	37	1	-6	-2	-1	0	1111
15	15	1	-1	-4	1	0	370
7	7	1	3	1	1	0	123
0	0	1	3	-4	1	0	41
1	4	-1	0	-3	-1	0	14
0	1	0	2	-2	2	0	4.6
-1	1	2	3	-1	2	0	1.5
0	0	0	0	0	0	0	0

Bliss sum : 281 (>0)

Δ Bliss values

T-3764518 (nM)

200	40	8	1.6	0.32	0.064	0	Vacuolin-1 (nM)
28	27	12	-3	-5	-2	0	30000
30	26	12	0	-1	-2	0	10000
25	20	-5	4	2	2	0	3333
24	22	-6	3	3	4	0	1111
24	20	-9	2	2	5	0	370
21	18	-2	4	6	5	0	123
16	15	4	6	10	6	0	41
10	8	3	8	8	5	0	14
6	4	2	9	10	8	0	4.6
1	0	0	4	6	6	0	1.5
0	0	0	0	0	0	0	0

Bliss sum : 472 (>0)

Δ Bliss values

T-3764518 (nM)

200	40	8	1.6	0.32	0.064	0	Hydroxychloroquine (nM)
41	41	18	-4	-3	3	0	30000
13	13	6	1	-2	1	0	10000
3	5	2	4	4	5	0	3333
8	6	4	4	4	8	0	1111
4	2	4	5	5	9	0	370
2	3	2	5	6	7	0	123
1	2	3	6	6	6	0	41
0	0	2	6	5	7	0	14
0	1	3	3	5	6	0	4.6
-1	1	2	3	5	7	0	1.5
0	0	0	0	0	0	0	0

Bliss sum : 317 (>0)

C

Δ Bliss values

T-3764518 (nM)

compound 7a (nM)	200	40	8	1.6	0.32	0.06	0
30000	-56	-57	-45	-2	-2	0	0
10000	-58	-59	-47	-6	-4	-2	0
3333	-54	-57	-45	-3	-1	-2	0
1111	-53	-56	-51	-7	-4	0	0
370	-49	-54	-108	-11	-5	-1	0
123	-26	-31	-90	-77	-30	2	0
41	4	1	-8	-53	-11	50	0
14	14	14	11	33	35	22	0
4.6	-6	-7	-20	-13	-8	-4	0
1.5	-2	-4	-14	-6	-3	0	0
0	0	0	0	0	0	0	0

Bliss sum : -1127 (<0)

Δ Bliss values

T-3764518 (nM)

GSK 2194069 (nM)	200	40	8	1.6	0.32	0.06	0
30000	-51	-48	-32	-3	-6	-5	0
10000	-48	-50	-39	-1	-2	-1	0
3333	-44	-50	-37	3	2	-1	0
1111	-46	-51	-46	-6	-3	-5	0
370	-46	-53	-45	-6	-3	-5	0
123	-43	-50	-44	-5	-2	-2	0
41	-42	-50	-45	-7	-6	-5	0
14	-41	-50	-45	-6	-4	-3	0
4.6	-40	-49	-44	-7	-3	-4	0
1.5	-34	-44	-41	-5	-3	-3	0
0	0	0	0	0	0	0	0

Bliss sum : -1458 (<0)

Δ Bliss values

T-3764518 (nM)

AZD8055 (nM)	200	40	8	1.6	0.32	0.06	0
30000	-53	-54	-48	-15	-7	-7	0
10000	-49	-49	-42	-13	-3	-1	0
3333	-42	-42	-35	-9	-2	-2	0
1111	-38	-39	-32	-7	0	-1	0
370	-35	-34	-31	-8	-2	-2	0
123	-30	-32	-26	-4	-1	2	0
41	-25	-26	-19	-1	3	1	0
14	-14	-15	-8	3	6	3	0
4.6	3	3	15	36	37	35	0
1.5	11	12	25	49	51	46	0
0	0	0	0	0	0	0	0

Bliss sum : -560 (<0)

Δ Bliss values

T-3764518 (nM)

Bax channel blocker (nM)	200	40	8	1.6	0.32	0.06	0
30000	-2	-2	0	0	1	0	0
10000	-1	-1	0	0	0	0	0
3333	7	7	13	21	8	-21	0
1111	20	22	15	-3	-1	3	0
370	13	12	5	3	-1	4	0
123	7	5	2	-2	-3	2	0
41	4	6	6	-3	-3	2	0
14	3	3	2	-4	-6	0	0
4.6	2	1	-2	-4	-7	-2	0
1.5	4	0	-1	-1	-3	3	0
0	0	0	0	0	0	0	0

Bliss sum : 131 (>0)

Δ Bliss values

T-3764518 (nM)

STA5326 (nM)	200	40	8	1.6	0.32	0.06	0
30000	-5	-6	-10	-7	-5	-6	0
10000	5	5	-3	-6	-5	-3	0
3333	11	10	4	-4	-2	-1	0
1111	11	12	13	-8	-4	-1	0
370	13	13	15	-6	-3	-1	0
123	13	13	16	-8	-5	-4	0
41	16	17	18	-9	-6	-3	0
14	17	15	13	-11	-7	-4	0
4.6	16	14	1	-9	-5	-1	0
1.5	14	11	-6	-7	-4	-2	0
0	0	0	0	0	0	0	0

Bliss sum : 132 (>0)

D

HT-15

Δ Bliss values

T-3764518 (nM) (columns) × compound 7a (nM) (rows)

compound 7a (nM)	200	40	8	1.6	0.32	0.06	0
30000	-69	-69	-62	-23	-5	-3	0
10000	-65	-61	-56	-23	-7	-4	0
3333	-53	-51	-48	-18	-5	-1	0
1111	-41	-39	-38	-13	-3	-2	0
370	-27	-25	-26	-5	-3	2	0
123	-21	-17	-18	-1	4	4	0
41	-11	-9	-11	2	0	4	0
14	-8	-5	-6	3	-1	3	0
4.6	-4	-3	-2	-1	-1	2	0
1.5	-5	-2	-3	-3	-4	1	0
0	0	0	0	0	0	0	0

Bliss sum : -955 (<0)

Δ Bliss values

T-3764518 (nM) (columns) × Bax channel blocker (nM) (rows)

Bax channel blocker (nM)	200	40	8	1.6	0.32	0.06	0
30000	0	0	0	0	0	0	0
10000	0	0	0	0	0	0	0
3333	1	1	1	3	3	3	0
1111	13	14	18	18	4	3	0
370	10	10	15	4	2	2	0
123	5	6	7	0	1	0	0
41	2	3	6	0	3	2	0
14	2	3	5	2	4	2	0
4.6	1	2	2	2	1	3	0
1.5	0	-1	2	1	1	1	0
0	0	0	0	0	0	0	0

Bliss sum : 194(>0)

HT-29

Δ Bliss values

T-3764518 (nM) (columns) × compound 7a (nM) (rows)

compound 7a (nM)	200	40	8	1.6	0.32	0.06	0
30000	-85	-83	-78	-31	-7	-4	0
10000	-77	-78	-72	-31	-4	-6	0
3333	-75	-73	-69	-27	-3	0	0
1111	-69	-67	-64	-29	-5	-1	0
370	-45	-44	-58	-24	-2	0	0
123	-35	-36	-50	-27	-2	2	0
41	-17	-19	-27	-19	0	0	0
14	-16	-16	-19	-19	-2	1	0
4.6	-12	-11	-15	-19	-4	-2	0
1.5	-8	-8	-12	-10	-2	2	0
0	0	0	0	0	0	0	0

Bliss sum : -1616 (<0)

Δ Bliss values

T-3764518 (nM) (columns) × Bax channel blocker (nM) (rows)

Bax channel blocker (nM)	200	40	8	1.6	0.32	0.06	0
30000	0	0	0	0	0	0	0
10000	0	0	0	0	0	0	0
3333	4	4	5	14	15	4	0
1111	22	23	28	24	-5	-1	0
370	16	17	20	8	0	4	0
123	12	11	16	10	0	1	0
41	8	7	11	12	3	5	0
14	5	6	7	11	1	1	0
4.6	3	4	3	6	0	2	0
1.5	1	1	-1	1	-2	1	0
0	0	0	0	0	0	0	0

Bliss sum : 349(>0)

SW620

Δ Bliss values

T-3764518 (nM) (columns) × compound 7a (nM) (rows)

compound 7a (nM)	200	40	8	1.6	0.32	0.06	0
30000	-63	-66	-61	-30	5	3	0
10000	-62	-64	-59	-29	2	-1	0
3333	-61	-63	-59	-31	4	3	0
1111	-62	-63	-64	-30	1	1	0
370	-53	-55	-63	-32	4	1	0
123	-40	-46	-53	-39	-1	-3	0
41	-24	-24	-32	-37	0	2	0
14	-19	-19	-19	-27	0	2	0
4.6	-14	-15	-16	-15	1	4	0
1.5	-12	-12	-12	-8	-2	-1	0
0	0	0	0	0	0	0	0

Bliss sum : -1530 (<0)

Δ Bliss values

T-3764518 (nM) (columns) × Bax channel blocker (nM) (rows)

Bax channel blocker (nM)	200	40	8	1.6	0.32	0.06	0
30000	0	0	0	0	0	0	0
10000	0	0	0	0	0	0	0
3333	0	0	0	0	0	0	0
1111	29	29	34	26	3	6	0
370	16	17	21	24	4	6	0
123	8	10	12	19	1	3	0
41	6	7	10	17	4	7	0
14	4	5	5	11	2	4	0
4.6	1	1	4	5	1	3	0
1.5	2	1	1	6	5	6	0
0	0	0	0	0	0	0	0

Bliss sum : 386(>0)
