# Supplementary material for: Feedback activation of AMPK-mediated autophagy acceleration is a key resistance mechanism against SCD1 inhibitor-induced cell growth inhibition
Source: PLoS One. 2017 Jul 13;12(7):e0181243. doi: 10.1371/journal.pone.0181243 (PMC5509324; doi:10.1371/journal.pone.0181243)
Supplement: S4 Fig — HCT-116 cells were treated with DMSO or T-3764518 for 24 h, and gene expression levels were analyzed via Human Genome U133 Plus 2.0 Array. Fold-increases for each gene in SCD1-WT cells treated with T-3764518 and SCD1-KO cells treated with DMSO relative to SCD1-WT cells treated with DMSO are shown. (PDF) [file pone.0181243.s004.pdf]

Symbol	Full name / Annotation	Fold increase	
		T-3764518 / DMSO	SCD1-KO / SCD1-WT
SCD	stearoyl-CoA desaturase (delta-9-desaturase)	5.3	1.8
FASN	fatty acid synthase	2.2	2.1
MAP1LC3B	microtubule associated protein 1 light chain 3 beta	2.9	2.4
