# Supplementary material for: Feedback activation of AMPK-mediated autophagy acceleration is a key resistance mechanism against SCD1 inhibitor-induced cell growth inhibition
Source: PLoS One. 2017 Jul 13;12(7):e0181243. doi: 10.1371/journal.pone.0181243 (PMC5509324; doi:10.1371/journal.pone.0181243)
Supplement: S1 Text — (DOCX) [file pone.0181243.s005.docx]

**Supporting Information**

**Materials and methods**

**Cell culture**

HCT-15, HT-29, and SW-620 cells were purchased from American Type Culture Collection and cultured in RPMI medium supplemented with 10% fetal bovine serum, and 1× penicillin/streptomycin at 37 °C and 5% CO_2_.

**Cell proliferation assay using measurement of cellular DNA contents**

HCT-116 cells were seeded at 5000 cells/well in 384-well black/clear plates (Corning). Twenty-four hours after cell seeding, indicated concentration of test compounds with or without T-3764518 were added to cells. After 72 h, cell proliferation was evaluated using a CyQUANT^®^ Direct Cell Proliferation Assay (Thermo Fisher Scientific) according to the manufacturer’s instructions. Plates were read on an EnVision Multilabel Reader (PerkinElmer) with 485 nm excitation, and 535 nm emission using a bottom-read mode. Wells containing no test compound and wells with no cells were used as 0% and 100% growth inhibition controls, respectively. Data were then used for Bliss sum analysis and calculation of Bliss sum scores.

**Cell proliferation assay using measurement of cellular ATP contents**

HCT-15, HT-29, and SW-620 cells were seeded at 5000 cells/well in 384-well black plates (Corning). Twenty-four hours after cell seeding, serially diluted test compounds and T-3764518 were to cells. After 72 h, cell viability was measured using a CellTiter-Glo^®^ Luminescent Cell Viability Assay (Promega) according to the manufacturer’s instructions with an EnVision Multilabel Reader (PerkinElmer). Wells containing no test compound and wells with no cells were used as 0% and 100% growth inhibition controls, respectively. Data were then used for Bliss sum analysis and calculation of Bliss sum scores.

**siRNA transfection and cell proliferation assay**

To silence AMPK, HCT-116 cells were transfected with siRNAs targeting PRKAA1 (Silencer^®^ Select Pre-designed siRNA; s100, s101, and s102) and PRKAA2 (s11056, s11057, and s11058) using RNAiMAX transfection reagent (Thermo Fisher Scientific). Transfection was performed using the reverse transfection method according to the manufacturer’s instructions. Negative control No. 1 siRNA (Thermo Fisher Scientific) was used as a control. Twenty-four hours after siRNA transfection, cells were treated with T-3764518 in a dose dependent fashion. After 72 h, cell viability was measured using a CellTiter-Glo^®^ Luminescent Cell Viability Assay. To test the knockdown of AMPK mRNA, cell lysates were prepared using a CellAmp Direct RNA Prep Kit for RT-PCR (Takara), and quantitative PCR was performed using a One Step PrimeScript RT-PCR Kit (Takara). TaqMan^®^ Gene Expression Assays (Thermo Fisher Scientific) were used for the following genes: PRKAA1 (Hs01562315_m1), and PRKAA2 (Hs00178903_m1).
